# EDTA-Reduction of Water to Molecular Hydrogen Catalyzed by Visible-Light-Response TiO_2_-Based Materials Sensitized by Dawson- and Keggin-Type Rhenium(V)-Containing Polyoxotungstates

**DOI:** 10.3390/ma3020897

**Published:** 2010-02-02

**Authors:** Chika Nozaki Kato, Kazunobu Hara, Masao Kato, Hidekuni Amano, Konomi Sato, Yusuke Kataoka, Wasuke Mori

**Affiliations:** 1 Department of Chemistry, Faculty of Science, Shizuoka University, 836 Ohya, Suruga-ku, Shizuoka 422-8529, Japan; 2 Department of Chemistry, Faculty of Science, Kanagawa University, Tsuchiya 2946, Hiratsuka, Kanagawa 259-1293, Japan

**Keywords:** polyoxometalate, rhenium, titanium dioxide, hydrogen evolution, visible light

## Abstract

The synthesis and characterization of a Keggin-type mono-rhenium(V)- substituted polyoxotungstate are described. The dimethylammonium salt [Me_2_NH_2_]_4_[PW_11_Re^V^O_40_] was obtained as analytically pure homogeneous black-purple crystals by reacting mono-lacunary Keggin polyoxotungstate with [Re^IV^Cl_6_]^2-^ in water, followed by crystallization from acetone at ca. 5 °C. Single-crystal X-ray structural analysis of [PW_11_Re^V^O_40_]^4-^ revealed a monomeric structure with overall *T_d_* symmetry. Characterization of [Me_2_NH_2_]_4_[PW_11_Re^V^O_40_] was also accomplished by elemental analysis, magnetic susceptibility, TG/DTA, FTIR, UV-vis, diffuse reflectance (DR) UV-vis, and solution ^31^P-NMR spectroscopy. Furthermore, [PW_11_Re^V^O_40_]^4-^ and the Dawson-type dirhenium(V)-oxido-bridged polyoxotungstate [O{Re^V^(OH)(α_2_-P_2_W_17_O_61_)}_2_]^14-^ were supported onto anatase TiO_2_ surface by the precipitation methods using CsCl and Pt(NH_3_)_4_Cl_2_. With these materials, hydrogen evolution from water in the presence of EDTA·2Na (ethylenediamine tetraacetic acid disodium salt) under visible light irradiation (≥400 nm) was achieved.

## 1. Introduction

Molecular hydrogen is known to be a clean-burning fuel free of CO_2_ emissions; it is considered a promising candidate to mitigate the current energy problems [1,2]. The development of efficient photocatalysts for hydrogen production from water has attracted much attention in the fields of solar light energy utilization and storage; Honda and Fujishima were the first to demonstrate a photo-electrochemical cell consisting of a TiO_2_ photo-anode and Pt cathode to decompose water into hydrogen and oxygen under ultraviolet (UV) irradiation with an external bias [3]. The design and preparation of new photocatalysts that are responsive in a similar manner to visible light are a key target for utilizing solar energy for hydrogen production. There have been several attempts to develop efficient photocatalysts that work under visible light irradiation: e.g., a chemically modified *n*-type TiO_2_ by controlled combustion of Ti metal in a neutral gas flame (this material absorbs light at wavelengths below 535 nm) [4]; visible-light-responsive TiO_2_ thin films prepared by a radio-frequency magnetron sputtering deposition method [5]; NiO_x_-promoted (partly oxidized nickel) In_0.9_Ni_0.1_TaO_4_ [6]; (AgIn)_x_Zn_2(1-x)_S_2_ [7]; (Ga_1-x_Zn_x_)(N_1-x_O_x_) with RuO_2_, transition metal mixed-oxides consisting of Cr, and noble-metal/Cr_2_O_3_ (core/shell) nanoparticles as co-catalysts [8]. These materials are powerful photocatalysts for water splitting under visible light irradiation; however, research on using other materials as visible-light-responsive photocatalysts still holds considerable interest.

Polyoxometalates (POMs) have attracted considerable attention because of their extreme versatility and unique range properties; these include catalytic and biological activities and/or photochemical, electrochromic, and magnetic properties [9,10,11,12]. Recently, it is known that the POMs-supported TiO_2_ and/or zeolite materials show higher activities for various photoreactions under visible light irradiation [13,14,15,16,17]. We also succeeded in developing a TiO_2_-based visible-light-responsive photocatalyst: a Dawson-type dirhenium(V)-oxido-bridged POM [O{Re^V^(OH)(α_2_-P_2_W_17_O_61_)}_2_]^14-^ (**1**) was grafted onto TiO_2_ through electrostatic interaction using a silane coupling reagent with cationic quaternary ammonium groups. The material showed activity for hydrogen evolution from water vapor under visible light irradiation (≥400 and ≥420 nm); however, the surface silane coupling reagent was decomposed by the visible light irradiation [18].

**Figure 1:**
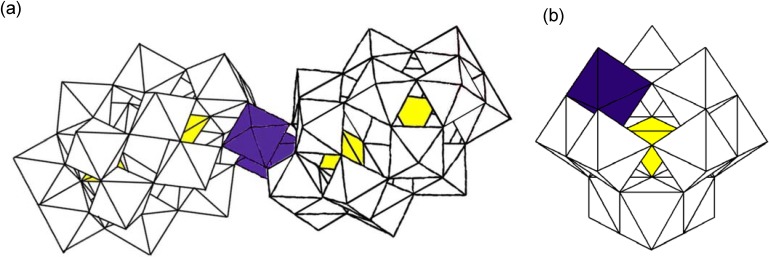
Polyhedral representation of (a) [O{Re^V^(OH)(α_2_-P_2_W_17_O_61_)}_2_]^14-^ (**1**) and (b) [PW_11_Re^V^O_40_]^4-^ (**2**). The one and two rhenium groups are represented by the purple octahedra. The WO_6_ and internal PO_4_ groups are represented by white octahedra and yellow tetrahedra, respectively.

In this study, we focused on using the Keggin-type mono-rhenium(V)-substituted POM [PW_11_Re^V^O_40_]^4-^ (**2**) as a sensitizer to investigate the influence of molecular structures of rhenium(V)-containing polyoxometalates for hydrogen evolution from water under visible light irradiation. The potassium and tetra-*n*-butylammonium salts of **2** have already been reported; however, the X-ray crystal structure of **2** has never been clarified [19,20]. In this paper, we synthesized the dimethylammonium salt of **2**, [Me_2_NH_2_]_4_[PW_11_Re^V^O_40_] (**Me_2_NH_2_-2**), by a different method, and characterized it using X-ray crystallography, elemental analysis, magnetic susceptibility, TG/DTA, FTIR, UV-vis, diffuse reflectance (DR) UV-vis, and solution ^31^P-NMR spectroscopy. The polyoxoanions **1** and **2** were then supported onto anatase TiO_2_ surface by the precipitation methods using CsCl and Pt(NH_3_)_4_Cl_2_ with some loadings. The photocatalytic activities of these materials were demonstrated for hydrogen evolution from water in the presence of EDTA·2Na (ethylenediamine tetraacetic acid disodium salt) under visible light irradiation (≥400 nm). The polyhedral representations of polyoxoanions **1** and **2** are shown in Figure 1.

## 2. Experimental Section

### 2.1. Materials

The potassium salt of **1**, K_14_[O{Re^V^(OH)(α_2_-P_2_W_17_O_61_)}_2_]·21H_2_O (**K-1**), was synthesized by the published method [18]. The mono-lacunary Keggin POM K_7_[PW_11_O_39_]·11H_2_O was synthesized by the published method, and was characterized by ^31^P-NMR, TG/DTA, and FTIR measurements [21]. The number of solvated water molecules for K_7_[PW_11_O_39_]·11H_2_O was determined by TG/DTA analysis. K_2_[Re^IV^Cl_6_] was purified by the reprecipitation from water/ethanol. Other reagents and solvents were obtained and used as received from commercial sources.

### 2.2. Instrumentation/Analytical Procedures

Elemental analyses were carried out by Mikroanalytisches Labor Pascher (Remagen, Germany). The samples were dried at room temperature under 10^-3^–10^-4^ torr overnight before analysis. Microanalyses for Re and P were specially ordered for POMs-supported TiO_2_ samples. Infrared spectra were recorded with a Jasco 4100 FTIR spectrometer on KBr disks at room temperature. Thermogravimetric (TG) and differential thermal analysis (DTA) data were acquired using a Rigaku Thermo Plus 2 series TG/DTA TG 8120. TG/DTA measurements were performed in air with the temperature increasing at a rate of 4 °C/min between R. T. and 500 °C. The ^31^P-{^1^H} NMR (161.70 MHz) spectra in solution were recorded in 5-mm outer diameter tubes with a JEOL ECA-600 NMR spectrometer. The ^31^P-{^1^H}-NMR spectra were referenced to an external standard of 85% H_3_PO_4_ in a sealed capillary. Chemical shifts were reported as negative on the *δ* scale with resonances upfield of H_3_PO_4_ (*δ* 0). Solution and diffuse reflectance (DR) UV-vis spectra were recorded on a Jasco V-570 spectrophotometer. For the DR UV-vis measurement, a Jasco diffuse-reflectance attachment was equipped. The positions of sharp bands were automatically determined by software of UV-vis spectrometer, and those of broad bands were picked up at the highest values in the ASCII files. Magnetic susceptibility of **Me_2_NH_2_-2** was evaluated using the Gouy method at Kanagawa University. The measurement was carried out at 25 °C.

### 2.3. Synthesis and Characterization of ***Me_2_NH_2_-2***

A mixture of K_2_ReCl_6_ (0.157 g, 0.33 mmol) and K_7_[PW_11_O_39_]·11H_2_O (1.0 g, 0.33 mmol) in 20 mL water was stirred for 1 h at 25 °C. The resulting dark purple-black solution was filtered through a folded filter paper (Whatman #5). Solid Me_2_NH_2_Cl (1.0 g, 12.26 mmol) was added to the dark purple-black filtrate at 25 °C. After stirring for 15 min, a purple-black precipitate was formed. This precipitate was collected on a membrane filter (JG 0.2 μm) and washed with ethanol (90 mL). At this stage, the product dimethylammonium salt was obtained in 0.14 g yield. The product (0.080 g) was dissolved in 50 mL acetone at room temperature, which was followed by filtering through a folded filter paper (Whatman #5). The purple-black solution was then allowed to evaporate slowly in a refrigerator at ca. 5 °C. After a few weeks, purple-black granular crystals were formed. The crystals were obtained in 36.1% yield (0.029 g scale) based on [(CH_3_)_2_NH_2_]_4_[PW_11_ReO_40_]. The obtained product was soluble in dimethylsulfoxide and slightly soluble in acetone, water, methanol, and ethanol. Elemental analysis: Found (calcd.) for [(CH_3_)_2_NH_2_]_4_[PW_11_ReO_40_] = H_32_C_8_N_4_O_40_P_1_Re_1_W_40_: H, 0.99% (1.05%); C, 3.40% (3.14%); N, 1.60% (1.83%); P, 0.96% (1.01%); Re, 5.80% (6.08%); W, 65.8% (66.01%). TG/DTA under atmospheric conditions: a weight loss of 6.27% with an exothermic point at 421.5 ºC was observed in the temperature range 64–500 ºC; the weight loss was due to the decomposition of Me_2_NH_2_^+^ ions (calculated value for 4Me_2_NH_2_^+^ ions was 6.02%). No weight loss due to the solvated water molecules was observed. Infrared spectrum (cm^-1^): 1076, 1020 (*ν_as_*(P-O_a_)), 972 (*ν_as_*(W-O_d_)), 885 (*ν_as_*(W-O_b_-W)), 800, 770 (*ν_as_*(W-O_c_-W)). ^31^P-NMR (in DMSO-*d*_6_, at 25 °C, referenced to 85% H_3_PO_4_): −14.93 ppm. UV-vis absorption (in DMSO, 1.00 × 10^-6^ M and 1.00 × 10^-4^ M): *λ* 264 nm (*ε* 49,950 M^-1^cm^-1^), *λ* 512 nm (*ε* 1,625 M^-1^cm^-1^), 741 nm (*ε* 786 M^-1^cm^-1^). DR UV-vis spectrum in the visible-light region: *λ* 526 nm, 688 nm. Magnetic moment: 1.28 B. M.

### 2.4. Preparation of ***1***- and ***2***-Supported TiO_2_ Materials by the Precipitation Method Using CsCl

For the preparation of **1**-supported TiO_2_ materials, anatase TiO_2_ support (2.0 g) was dispersed in 50 mL of water at 25 ºC. **K-1** (42.9 and 97.5 mg; 4.4 and 10.0 μmol of Re/g) was dissolved in 30 mL of water. The POM solution was then added to the TiO_2_ suspension. CsCl (414.9 and 942.5 mg; 1,232 and 2,800 μmol/g) was dissolved in 20 mL water and added to the mixtures. At this stage, 560 eq. of CsCl was required for the precipitation of polyoxoanion **1**. The elemental analysis results of Cs for the precipitate prepared by adding 560 eq. of CsCl to the aqueous solution of **1** was 16.9%; this showed that the formula of the obtained precipitate was Cs_14_[O{Re(OH)(α_2_-P_2_W_17_O_61_)}_2_]·22H_2_O (the calculated value was 16.91%). TG/DTA showed a weight loss of 3.93% with an endothermic point at 41.4 °C in the temperature range of 15.1–246 °C; the weight loss was due to the hydrated water molecules (calculated value for 22 H_2_O was 3.60%). After stirring for 2.5 h at 25 °C, the obtained purple-white products were collected by a Büchner funnel (Whatman #5), washed with water (30 mL × 3), and then dried in an oven at 50 °C overnight. Elemental analysis results for Re were 0.038% and 0.062%, respectively, which corresponded to 2.0 and 3.3 μmol of Re/g. These samples are abbreviated as **1**-Cs-TiO_2_(2.0) and **1**-Cs-TiO_2_(3.3), respectively. The DR UV-vis spectra in the visible light region of these materials showed sharp bands at 499 and 501 nm, respectively, which was assigned to the Re^V^ ➔ W^VI^ intervalence charge transfer (IVCT) band. In addition, a broad band at around 750 nm assigned to the *d-d* band of Re^V^ was observed for both samples (Figure S1).

For the **2**-supported TiO_2_ materials, **Me_2_NH_2_-2** (27.0 and 61.0 mg; 4.4 and 10.0 μmol of Re/g) was added to anatase TiO_2_ support (2.0 g) suspended in 300–550 mL of water at 25 ºC. CsCl (356 and 808 mg; 1056 and 2400 μmol/g) was dissolved in 20 mL water and added to the mixture. At this stage, 240 eq. of CsCl was required for the complete precipitation of **2**. The elemental analysis results of Cs for the precipitates prepared by adding 240 eq. of CsCl to the aqueous solution of **2** was 13.9%; this showed that the formula of the obtained precipitate was Cs_3.5_H_0.5_[PW_11_ReO_40_]·4H_2_O (the calculated value of Cs was 13.61%). TG/DTA showed a weight loss of 2.18% with an endothermic point at 59.2 °C in the temperature range of 17.4–168 °C; the weight loss was due to the hydrated water molecules (calculated value for 4 H_2_O was 2.11%), and no weight loss due to the decomposition of Me_2_NH_2_^+^ ions was observed. After stirring for 2 h at 25 °C, the obtained purple-white products were collected by a Büchner funnel (Whatman #5), washed with water (300 mL), and then dried in an oven at 50 °C overnight. Elemental analysis results of Re were 0.0069% and 0.042%, which corresponded to 0.37 and 2.3 μmol of Re/g, respectively. These samples are abbreviated as **2**-Cs-TiO_2_(0.37) and **2**-Cs-TiO_2_(2.3), respectively. The DR UV-vis spectrum in the visible light region of **2**-Cs-TiO_2_(2.3) showed a sharp band at 541 nm (Re^V^➔W^VI^ IVCT band) and a broad band at around 750 nm (*d-d* band of Re^V^), as shown in Figure S2a.

### 2.5. Preparation of ***1***- and ***2***-Supported TiO_2_ Materials by the Precipitation Method Using Pt(NH_3_)_4_Cl_2_

For the preparation of **1**-supported TiO_2_ materials, anatase TiO_2_ support (2.0 g) was dispersed in 50 mL of water at 25 ºC. Compound **K-1** (42.9, 97.5, and 146.2 mg; 4.4, 10.0, and 15.0 μmol of Re/g) was dissolved in 30 mL of water. The POM solution was then added to the TiO_2_ suspension. The mixture was stirred at 25 ºC overnight in the dark. Pt(NH_3_)_4_Cl_2_·H_2_O (15.6, 35.2, and 52.8 mg; 44, 100, and 150 μmol/g) was dissolved in 20 mL of water and added to the mixture. At this stage, 10 eq. of Pt(NH_3_)_4_Cl_2_·H_2_O was required for the precipitation of polyoxoanion **1**. The elemental analysis result of Pt for the precipitate prepared by adding 10 eq. of Pt(NH_3_)_4_Cl_2_·H_2_O to the aqueous solution of **1** was 12.2%; this showed that the formula of the obtained precipitate was [Pt(NH_3_)_4_]_6.5_K[O{Re(OH) (α_2_-P_2_W_17_O_61_)}_2_]·15H_2_O (11.77%). TG/DTA showed a weight loss of 2.57% with endothermic points at 30.9 and 64.0 °C in the temperature range of 15.9–180 °C; the weight loss was due to the hydrated water molecules (calculated value for 15 H_2_O was 2.51%). After stirring overnight at 25 °C, the obtained purple-white product was collected by a Büchner funnel (Whatman #5) and washed with water (30 mL × 3). The elemental analysis results for Re were 0.030%, 0.073%, and 0.105%, which corresponded to 1.6, 3.9, and 5.6 μmol of Re/g, respectively. These samples were abbreviated as **1**-Pt-TiO_2_(1.6), **1**-Pt-TiO_2_(3.9), and **1**-Pt-TiO_2_(5.6), respectively. The DR UV-vis spectra in the visible light region of these materials showed a sharp band at 505, 523, and 525 nm (Re^V^ ➔ W^VI^ IVCT band), respectively. In addition, a broad band was observed at around 750 nm (*d-d* band of Re^V^) for all samples (Figure S3).

For the **2**-supported TiO_2_ materials, **Me_2_NH_2_-2** (27.0 and 61.3 mg; 4.4 and 10.0 μmol of Re/g) dissolved in water (200 mL) was added to anatase TiO_2_ support (2.0 g) suspended in 50 mL of water at 25 ºC. Pt(NH_3_)_4_Cl_2_·H_2_O (62 and 141 mg; 176 and 400 μmol/g) was dissolved in 20 mL water and added to the mixture. At this stage, 20 eq. of [Pt(NH_3_)_4_]Cl_2_·H_2_O was required for the complete precipitation of **2**. The elemental analysis results of Pt for the precipitates prepared by adding 20 eq. of [Pt(NH_3_)_4_]Cl_2_·H_2_O to the aqueous solution of **2** was 12.0%; this showed that the formula of the obtained precipitate was [Pt(NH_3_)_4_]_2_[PW_11_ReO_40_] (the calculated value of Pt was 11.46%). TG/DTA observed no weight loss in the temperature range of 17.1–200 °C, showing that no hydrated water was contained. After stirring overnight at 25 °C, the obtained purple-white products were collected by a Büchner funnel (Whatman #5), washed with water (30 × 3 mL), and then dried in an oven at 50 °C overnight. Elemental analysis results of Re were 0.0011% and 0.0035%, which corresponded to 0.059 and 0.19 μmol of Re/g, respectively. These samples are abbreviated as **2**-Pt-TiO_2_(0.059) and **2**-Pt-TiO_2_(0.19), respectively. No clear bands were observed for **2**-Pt-TiO_2_(0.059) and **2**-Pt-TiO_2_(0.19) for their DR UV-vis spectra due to the low loadings.

### 2.6. Catalytic Reaction Experiments

The H_2_ evolution from water was carried out at 25 ºC. A mixture of catalyst (200 mg), water (10 mL), and EDTA·2Na (30 mM) was placed into a glass reaction vessel; this was connected to a Pyrex conventional closed gas circulation system (238.8 cm^3^). The photoreaction was started by light irradiation with a 500 W Xe lamp equipped with a cut-off filter (λ ≥400 nm). H_2_, O_2_, CO, and CH_4_ were analyzed by GC (TCD, Molecular Sieve 5A stainless columns), and water and CO_2_ were analyzed by GC (TCD, Porapak Q stainless columns): the samples were assigned after they were compared with authentic samples analyzed under the same conditions. Turnover number (TON) was calculated as 2[hydrogen evolved (mol/g of catalyst)]/[Re atoms (mol/g of catalyst)]. Turnover frequency (TOF) was calculated as [TON] / [reaction time (h)].

## 3. Results and Discussion

### 3.1. Synthesis and Characterization of [Me_2_NH_2_]_4_[PW_11_Re^V^O_40_] *(**Me_2_NH_2_-2**)*

The dimethylammonium salt of **2**, [Me_2_NH_2_]_4_[PW_11_Re^V^O_40_] (**Me_2_NH_2_-2**), was synthesized with a slight modification of the published method for the potassium salt of the Dawson-type dirhenium(V)-oxido-bridged POM K_14_[O{Re^V^(OH)(α_2_-P_2_W_17_O_61_)}_2_]·21H_2_O (**K-1**) [18]. The compound **Me_2_NH_2_-2** was formed by stirring a mixture of K_2_Re^IV^Cl_6_ and mono-lacunary Keggin POM [PW_11_O_39_]^7-^ in an aqueous solution under air at 25 °C; this was followed by the addition of excess Me_2_NH_2_Cl to form the dark purple-black precipitate. The unreacted Me_2_NH_2_Cl was completely removed by washing with ethanol.

The elemental analysis for compound **Me_2_NH_2_-2** had to be performed after drying at room temperature at 10^-3^–10^-4^ torr overnight. The result was consistent with the composition [(CH_3_)_2_NH_2_]_4_[PW_11_Re^V^O_40_]. The weight loss observed during the course of drying before analysis was 0.3% for **Me_2_NH_2_-2**; this suggested the absence of solvated or adsorbed water molecule. The TG/DTA measurements performed under atmospheric conditions showed a weight loss of 6.27% with an exothermic point; this value corresponds to four Me_2_NH_2_^+^ ions. No weight loss due to the solvated water molecules was observed.

**Figure 2:**
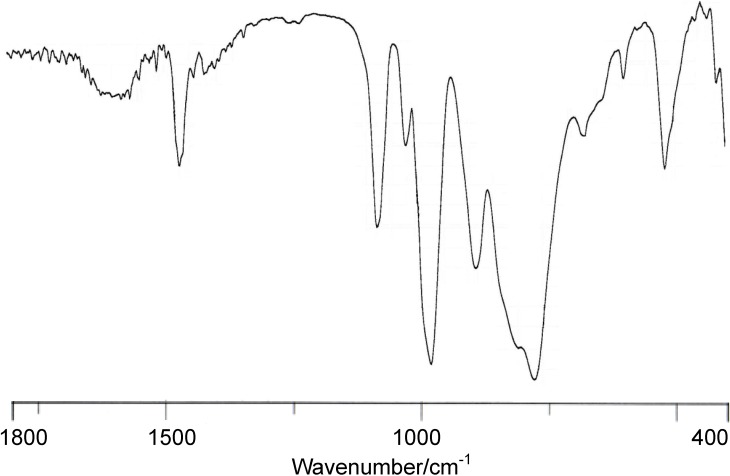
FTIR spectrum (as KBr disks) of **Me_2_NH_2_-2**.

The FTIR spectrum of compound **Me_2_NH_2_-2** that was measured on a KBr disk is shown in Figure 2. The positions of all bands (1076, 1020, 972, 885, 800, and 770 cm^-1^) in the polyoxoanion region of this compound are characteristic of polyoxoanion; however, they were different from those of [PW_12_O_40_]^4-^ (1080, 984, 893, and 808 cm^-1^) and [PW_11_O_39_]^7-^ (1086, 1043, 953, 903, 862, 810, and 734 cm^-1^). This suggests the coordination of rhenium atoms into the monovacant site of [PW_11_O_39_]^7-^.

The magnetic moment of **Me_2_NH_2_-2** was 1.28 B. M., which is in the range for Cs_2_[Re^V^OCl_5_] (1.0–2.0 B. M.) [22]. This value was smaller than the theoretical value (2.83 B. M.), suggesting that the rhenium(V) site in **Me_2_NH_2_-2** is weakly paramagnetic.

**Figure 3:**
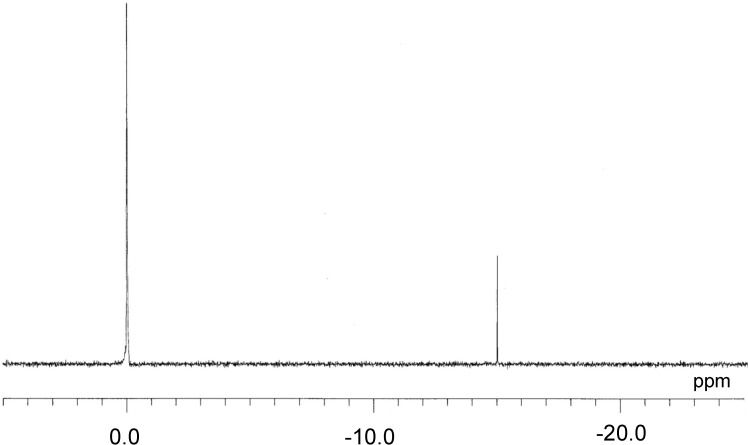
^31^P-NMR spectra in DMSO-*d*_6_ of **Me_2_NH_2_-2**. The resonance at 0.0 ppm is due to the external reference: 85% H_3_PO_4_.

The ^31^P-NMR spectrum in DMSO-*d*_6_ of **Me_2_NH_2_-2** before crystallization from acetone showed a clear single-line spectrum at *δ* = −14.93, as shown in Figure 3. The signal exhibited a shift from that of [PW_11_O_39_]^7-^ (*δ* = −10.12) and [PW_12_O_40_]^3-^ (*δ* = −14.67), indicating the complete coordination of rhenium atom into the monovacant site of [PW_11_O_39_]^7-^ and the high purity of **Me_2_NH_2_-2**. The ^31^P NMR spectrum in D_2_O of the potassium salt of **2** (*δ* = −15.1) was first reported by Pope *et al*. [20]. The ^31^P NMR spectrum of the crystalline sample for **Me_2_NH_2_-2** as crystallized from the acetone solution also showed the same chemical shifts (*δ* = −14.92) as those of the powder sample.

**Figure 4:**
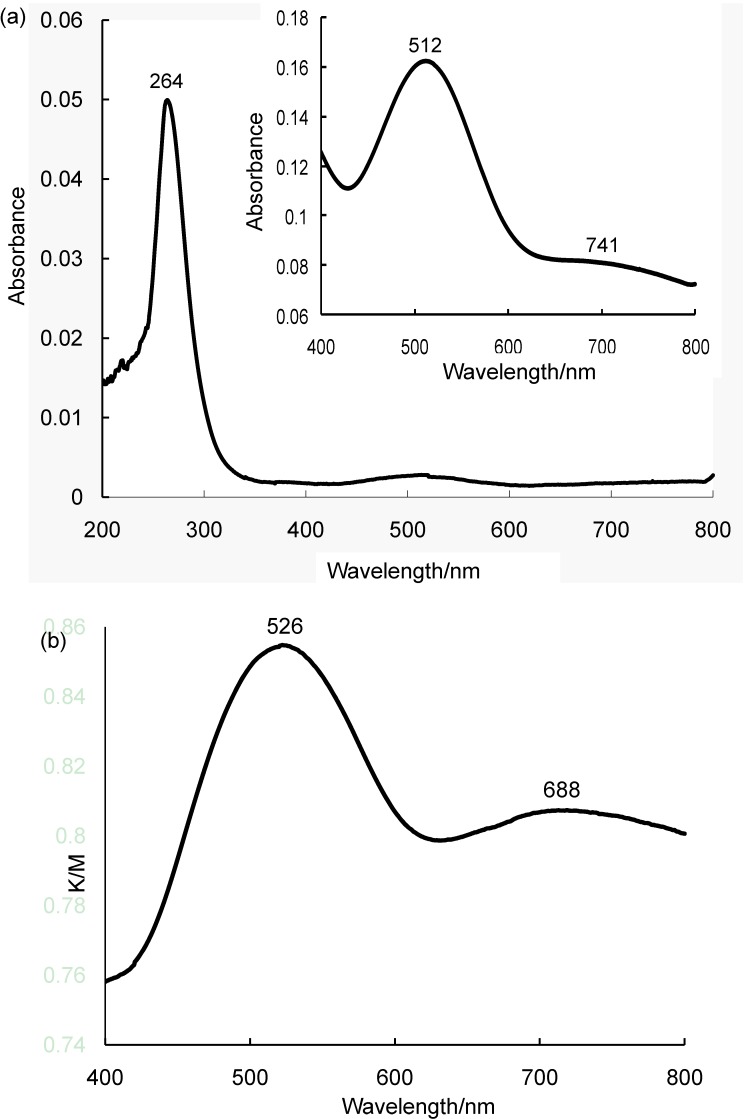
(a) UV-vis spectrum in DMSO of **Me_2_NH_2_-2** at 200–800 nm (1.0 × 10^-6^ M). Inset: 400–800 nm (1.0 × 10^-4^ M). (b) Diffuse reflectance UV-vis spectrum of **Me_2_NH_2_-2** at 400–800 nm.

The UV-vis spectrum of **Me_2_NH_2_-2** in DMSO showed three absorption bands at 264 (*ε* 49,950 M^-1^cm^-1^), 512 (*ε* 1625 M^-1^cm^-1^), and 741 nm (*ε* 786 M^-1^cm^-1^), as shown in Figure 4a. A large band at 264 nm was assigned to the charge transfer (CT) band of W-O, and two small bands at 512 and 741 nm were assigned to the Re^V^➔W^VI^ intervalence charge transfer (IVCT) band and *d-d* band of the rhenium(V) atom, respectively [19,20]. The positions of the two bands at 512 and 741 nm were similar to those of **K-1** (496 and 737 nm). The DR UV-vis spectrum of **Me_2_NH_2_-2** in the visible-light region also showed two bands at 526 and 688 nm due to the Re^V^➔W^VI^ IVCT band and *d-d* band of the rhenium(V) atom, respectively (Figure 4b). The positions of these bands were quite similar to those of compound **K-1** (532 and 686 nm).

The crystallization of **Me_2_NH_2_-2** for X-ray crystal analysis was performed by slow evaporation from acetone in the dark. X-ray crystallography revealed that **2** was a monomeric α-Keggin POM with *T_d_* symmetry, but the molecular structure of **2** was not determined because of the disorder of eleven tungsten(VI) atoms due to a highly symmetric space group; this is similar to the earlier cases of [W_9_ReO_32_]^5-^ [23] and [PW_11_(TiO_2_)O_39_]^5-^ [24].

### 3.2. Hydrogen Evolution from an Aqueous Solution Containing EDTA·2Na Catalyzed by ***1***-Supported TiO_2_ Materials under Visible Light Irradiation (≥400 nm)

**Figure 5:**
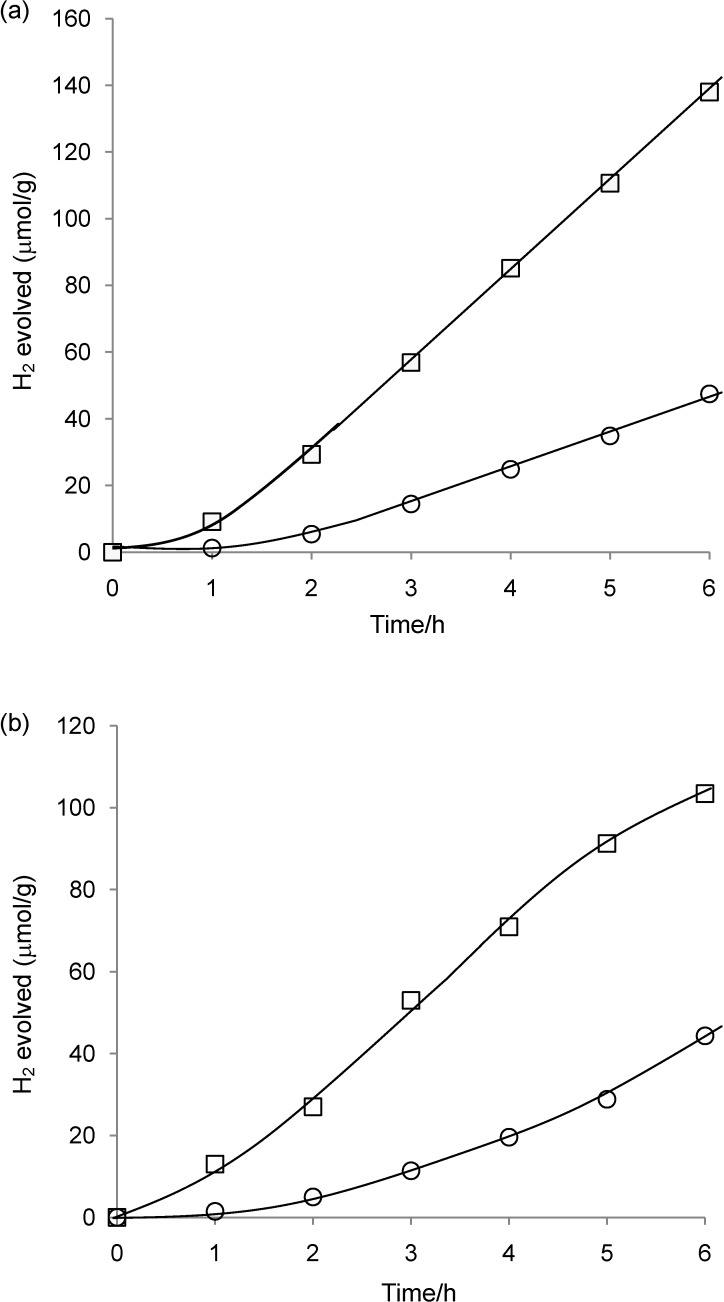
Time course for H_2_ evolution catalyzed by (a) **1**-Cs-TiO_2_(2.0) and (b) **1**-Cs-TiO_2_(3.3) under light irradiation (≥400 nm). The first and second runs are represented by circles (○) and squares (□), respectively. Reaction conditions: see Table 1.

**Figure 6:**
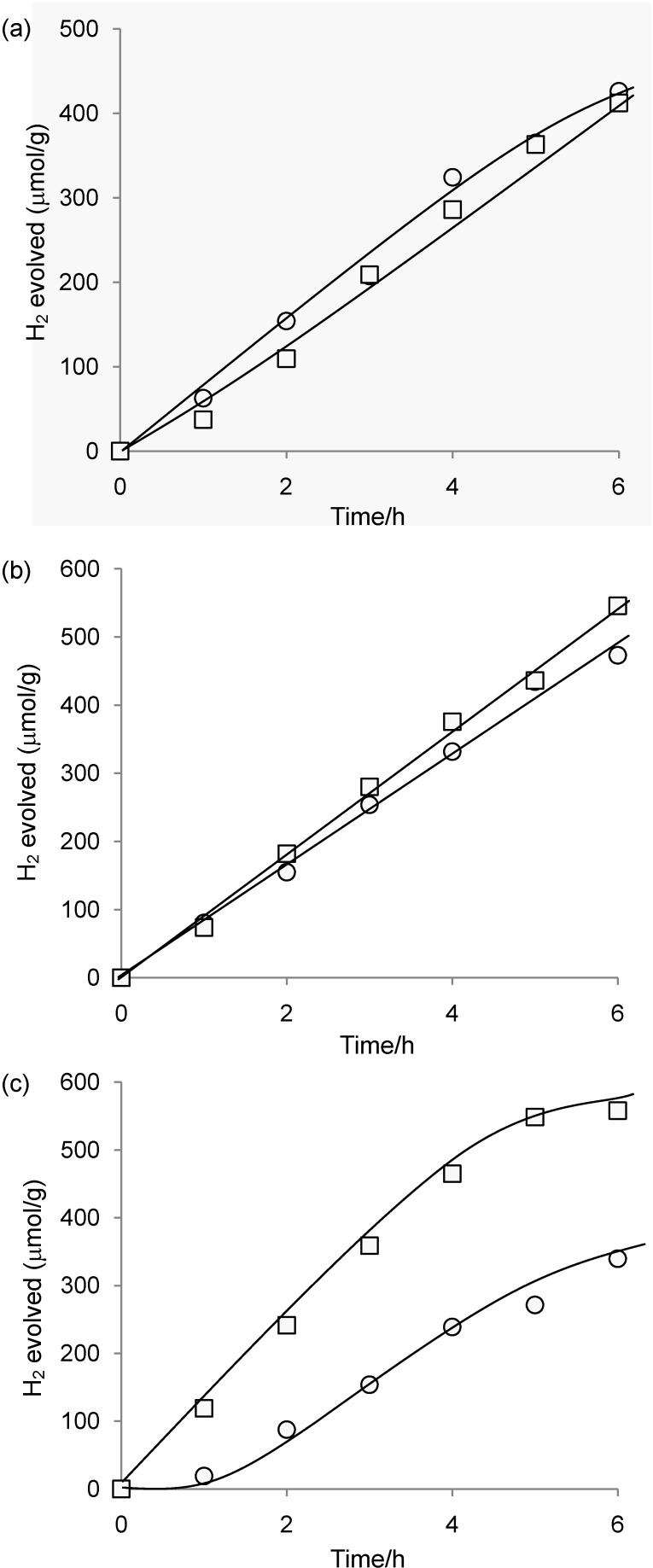
Time course for H_2_ evolution catalyzed by (a) **1**-Pt-TiO_2_(1.6), (b) **1**-Pt-TiO_2_(3.9), and (c) **1**-Pt-TiO_2_(5.6) under light irradiation (≥400 nm). The first and second runs are represented by circles (○) and squares (□), respectively. Reaction conditions: see Table 1.

We examined the hydrogen evolution from water in the presence of EDTA·2Na under light irradiation (≥400 nm), which was catalyzed by **1**-Cs-TiO_2_(2.0), **1**-Cs-TiO_2_(3.3), **1**-Pt-TiO_2_(1.6), **1**-Pt-TiO_2_(3.9), and **1**-Pt-TiO_2_(5.6) at 25ºC in a heterogeneous system; the results are summarized in Table 1. For the time course of H_2_ evolution in the first run, an induction period was observed for **1**-Cs-TiO_2_(2.0), **1**-Cs-TiO_2_(3.3), and **1**-Pt-TiO_2_(5.6), as shown in Figure 5 and Figure 6c. In particular, inactivation was observed for **1**-Pt-TiO_2_(5.6). In contrast, a linear increase in H_2_ with time was observed for **1**-Pt-TiO_2_(1.6) and **1**-Pt-TiO_2_(3.9), as shown in Figure 6a and Figure 6b. O_2_, CO_2_, CO, and CH_4_ were not detected under the present reaction conditions. The colors of these materials changed from white-purple to blue during the reactions; however, the blue-color disappeared and the photoreactions stopped when the visible light irradiation stopped. The pH of the solution changed from ca. 4.6 to ca. 6.2 after 6 h for all samples, suggesting that OH^-^ may have formed [25,26].

For **1**-Cs-TiO_2_(2.0) and **1**-Cs-TiO_2_(3.3), the amount of H_2_ after 1 h evolved was 1.24 and 1.48 μmol/g of catalyst, respectively. After 6 h, the amount of H_2_ evolved increased to 47.5 and 44.4 μmol/g, respectively (TON reached 48 and 27); however, the activities did not increase with the loading of **1**. In the control experiments, polyoxoanion **1** dissolved in aqueous EDTA solution showed no reaction, and hydrogen was slightly detected when catalyzed by TiO_2_; the sample was washed with a large amount of water and dried at 200 °C overnight. On contrary, polyoxoanion **1** (4.4 μmol of Re/g) dissolved in aqueous EDTA solution showed 221.4 μmol/g (TON = 101) of hydrogen evolution in the presence of TiO_2_. Thus, the polyoxoanion **1** leached into the aqueous solution is not negligible in the presence of TiO_2_.

To determine whether the surface polyoxoanion **1** leached into the solution during the photoreactions, 200 mg of **1**-Cs-TiO_2_(2.0) and **1**-Cs-TiO_2_(3.3) were irradiated in a 30 mM EDTA·2Na solution (10 mL) for 6 h; these mixtures were then filtered. The elemental analysis results for Re revealed that the loadings of the obtained solids after the first visible-light irradiation were 0.32 and 0.15 μmol/g, respectively; this suggested that 4.5%–16% of the surface polyoxoanion **1** remained on the TiO_2_ surface. In the recycle experiments, where the obtained solids after the first irradiation were used as catalysts for the second run, the amount of hydrogen after 6 h evolved was 138.0 and 103.5 μmol/g, respectively, which was 2–3 times larger than results for the first run. TON reached 863 and 1380, which were 18–51 times higher than in the results for the first run. As a control experiment, PW_12_O_40_^3-^-supported TiO_2_ material was precipitated using CsCl, where the loading of PW_12_O_40_^3-^ was 9.2 μmol/g; 89.9 μmol/g·h of hydrogen evolution resulted after 6 h (TON = 20). This result indicated that the hydrogen evolution occurred without the rhenium(V) site in polyoxoanion under the present conditions. However, the recycle experiment of the PW_12_O_40_^3-^-supported TiO_2_ material in the second run showed 87.3 μmol/g of hydrogen evolution, and TON as calculated on the basis of the elemental analysis results for P after the first irradiation was 17 after 6 h; this was 51–81 times lower than the results for **1**-Cs-TiO_2_(2.0) and **1**-Cs-TiO_2_(3.3) in the second run.

**Table 1:** Hydrogen evolution from water catalyzed by **1**-supported TiO_2_ materials under visible light irradiation. ^[a]^

Entry	Catalyst	Reaction time [h]	Recycle	H_2_ [μmol/g]	TON^[b]^
1	**1**-Cs-TiO_2_(2.0)	1	1st run	1.24	–
		6		47.5	48
		1	2nd run	9.17	–
		6		138.0	863^[c]^
2	**1**-Cs-TiO_2_(3.3)	1	1st run	1.48	–
		6		44.4	27
		1	2nd run	13.0	–
		6		103.5	1380^[c]^
3	**1**-Pt-TiO_2_(1.6)	1	1st run	62.6	–
		6		426.1	533
		1	2nd run	37.3	–
		6		412.3	9062^[c]^
4	**1**-Pt-TiO_2_(3.9)	1	1st run	80.3	–
		6		473.1	243
		1	2nd run	73.7	–
		6		545.7	3307^[c]^
5	**1**-Pt-TiO_2_(5.6)	1	1st run	19.1	–
		6		339.7	121
		1	2nd run	118.9	–
		6		558.0	1313^[c]^

[a] Reaction conditions: water (10 mL), catalyst (200 mg), EDTA·2Na (30 mM), light (≥400 nm), 25 ºC.

[b] Turnover number (TON) was calculated as 2[H_2_ evolved (mol/g)] per [Re atoms (mol/g)].

[c] The concentration of Re atoms for **1**-supported TiO_2_ materials after the first visible-light irradiation (≥400 nm) was 0.32, 0.15, 0.091, 0.33, and 0.85 μmol/g, respectively.

To investigate the stability of polyoxoanion **1** during the photoreactions, polyoxoanion **1** dissolved in an aqueous solution containing EDTA·2Na was irradiated by visible light (≥400 nm) in the presence of TiO_2_ for 6 h and characterized by ^31^P-NMR spectroscopy. The ^31^P-NMR spectrum in D_2_O of **1** after visible light irradiation showed a set of signals at −11.87 and −12.86 ppm, which was similar to the values of the as-prepared polyoxoanion **1** (*δ* −12.06 and −13.05) [18]. This result suggested that the molecular structure of **1** dissolved in aqueous solution still remained after visible light irradiation in the presence of TiO_2_. The DR UV-vis spectra of **1**-Cs-TiO_2_(2.0) and **1**-Cs-TiO_2_(3.3) after the photoreactions are shown in Figure S4. The spectra for the two showed the Re^V^ ➔ W^VI^ IVCT band at 505 and 504 nm, respectively; these are the same values as those for the as-prepared materials, suggesting that the rhenium(V) species still remained after the photoreactions. Thus, the conditions of **1** in solution and solid state, e.g., concentration, dispersion, and interaction of **1** with TiO_2_ surface, might influence the activities in the second run.

For **1**-Pt-TiO_2_(1.6), **1**-Pt-TiO_2_(3.9), and **1**-Pt-TiO_2_(5.6), the amount of H_2_ evolved after 1 h was 62.6, 80.3, and 19.1 μmol/g of catalyst, respectively. After 6 h, the amount of H_2_ evolved reached 426.1, 473.1, and 339.7 μmol/g, respectively. TON was 533, 243, and 121; these values were 2.5–19.7 times higher than those of **1**-Cs-TiO_2_(2.0) and **1**-Cs-TiO_2_(3.3), showing that Pt enhanced the catalytic activities remarkably as reported for various types of photocatalysts [3,27]. The TOF of **1**-Pt-TiO_2_(1.6), **1**-Pt-TiO_2_(3.9), and **1**-Pt-TiO_2_(5.6) were 20–89 h^-1^, which were higher than those of the reported dye-sensitized TiO_2_ materials used in heterogeneous systems containing EDTA; e.g., platinum-loaded Langmuir-Blodgett film of viologen-linked porphyrin (720 > *λ* > 390; TOF = 0.8 h^-1^) [28], acid-restacked calcium niobate nanosheets sensitized by Ru(bpy)_2_ (4,4’-(PO_3_H_2_)_2_bpy)^2+^ (*λ* > 420 nm; TOF = 5.4 h^-1^) [29], and zinc porphyrin/Pt/TiO_2_ system (*λ* >520 nm; TOF = 20 h^-1^) [30]. However, the TON decreased with the loading of **1**, as observed for **1**-Cs-TiO_2_(2.2) and **1**-Cs-TiO_2_(3.3).

The leaching of the surface polyoxoanion **1** into the solution was also determined for **1**-Pt-TiO_2_(1.6), **1**-Pt-TiO_2_(3.9), and **1**-Pt-TiO_2_(5.6) under the same reaction conditions as those for **1**-Cs-TiO_2_(2.0) and **1**-Cs-TiO_2_(3.3). The elemental analysis results for Re revealed that the loadings of the obtained solids after the first visible-light irradiation were 0.091, 0.33, and 0.85 μmol/g, respectively; this suggested that 5.7%–15% of the surface polyoxoanion **1** remained on the TiO_2_ surface. The recycle experiments for the obtained solids showed hydrogen evolved after 6 h was 412.3, 545.7, and 558.0 μmol/g, respectively. TON reached 9062, 3307, and 1313, which were 10.9–17.0 times higher than the values in the first run. Although the total amounts of H_2_ evolved after 6 h somewhat increased with the loading of **1**, TON was observed to decrease in the second run. The DR UV-vis spectra of **1**-Pt-TiO_2_(1.6), **1**-Pt-TiO_2_(3.9), and **1**-Pt-TiO_2_(5.6) after the photoreactions are shown in Figure S5. The Re^V^➔W^VI^ IVCT bands for these materials were observed at 488, 500, and 500 nm; these bands were somewhat blue-shifted from those of the as-prepared materials, and the band shapes were broadened after the photoreactions.

### 3.3. Hydrogen Evolution from an Aqueous Solution Containing EDTA·2Na Catalyzed by ***2***-Supported TiO_2_ Materials under Visible Light Irradiation (≥400 nm)

**Figure 7:**
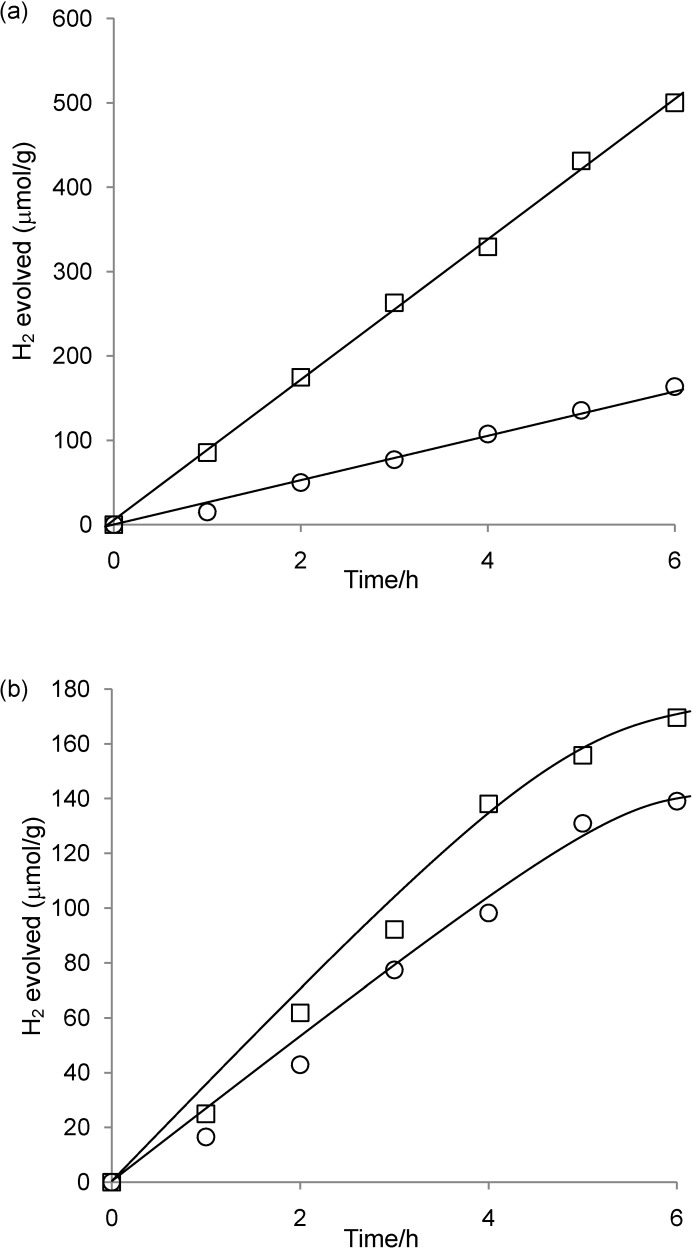
Time course for H_2_ evolution catalyzed by (a) **2**-Cs-TiO_2_(0.37) and (b) **2**-Cs-TiO_2_(2.3) under light irradiation (≥400 nm). The first and second runs are represented by circles (○) and squares (□), respectively. Reaction conditions: see Table 2.

**Figure 8:**
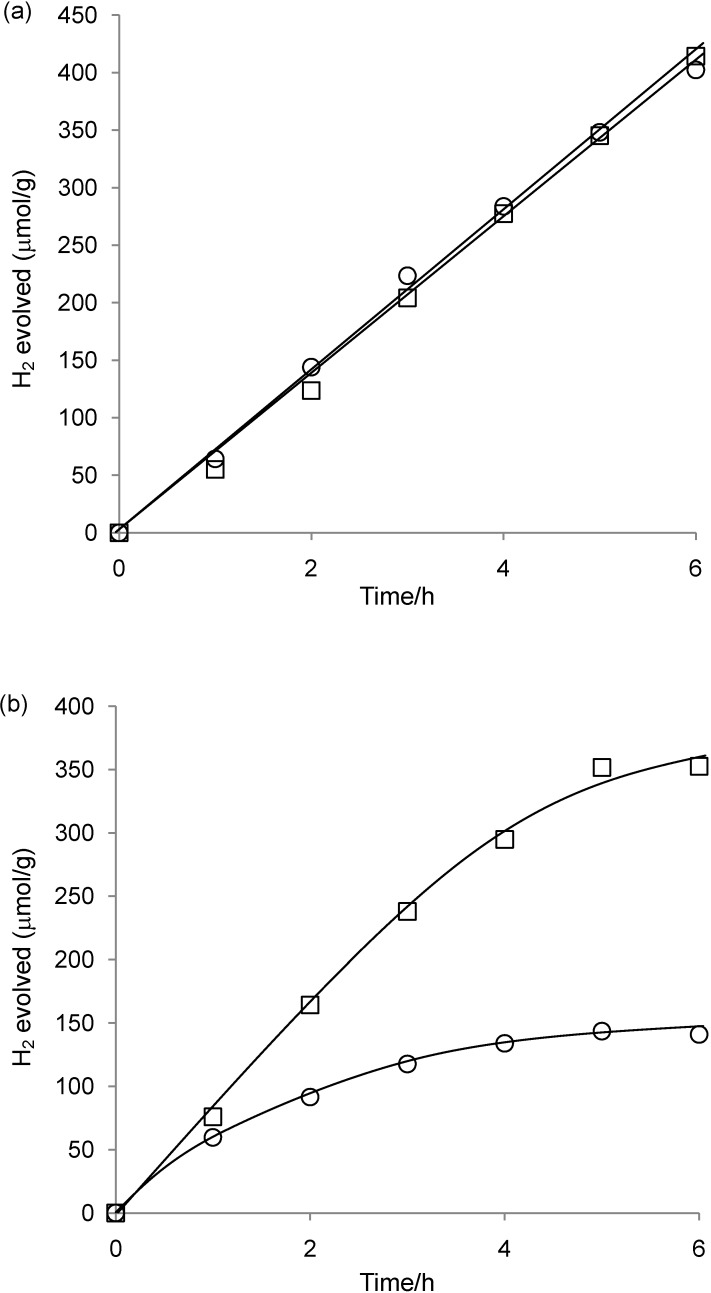
Time course for H_2_ evolution catalyzed by (a) **2**-Pt-TiO_2_(0.059) and (b) **2**-Pt-TiO_2_(0.19) under light irradiation (≥400 nm). The first and second runs are represented by circles (○) and squares (□), respectively. Reaction conditions: see Table 2.

We examined the hydrogen evolution from an aqueous solution containing EDTA·2Na under light irradiation (≥400 nm) that was catalyzed by **2**-Cs-TiO_2_ (0.37), **2**-Cs-TiO_2_ (2.3), **2**-Pt-TiO_2_ (0.059), and **2**-Pt-TiO_2_(0.19) at 25 ºC in a heterogeneous system; the results are summarized in Table 2. For the time course of H_2_ evolution in the first run, a linear increase in H_2_ with time was observed for **2**-Cs-TiO_2_ (0.37), **2**-Cs-TiO_2_ (2.3), and **2**-Pt-TiO_2_ (0.059) at the initial step; the increases are shown in Figure 7 and Figure 8. In contrast, significant inactivation was observed for **2**-Pt-TiO_2_ (0.19). The colors of these materials also changed from white-purple to blue during the reactions, and the pH of the solutions changed from ca. 4.7 to (5.9–6.1) after 6 h for all samples; this suggested that OH^-^ may have formed [25,26]. As a control experiment, **2** dissolved in aqueous solution showed no reaction under the present conditions.

For **2**-Cs-TiO_2_ (0.37) and **2**-Cs-TiO_2_ (2.3), the amount of H_2_ evolved after 1 h was 15.1 and 16.5 μmol/g of catalyst, respectively. After 6 h, the amounts of H_2_ evolved increased to 163.7 and 139.1 μmol/g, respectively. TON reached 885 and 121, which were 2.5–33 times higher than the values for **1**-Cs-TiO_2_ (2.0) and **1**-Cs-TiO_2_ (3.3). For the recycle experiments, 200 mg of **2**-Cs-TiO_2_ (0.37) and **2**-Cs-TiO_2_ (2.3) were irradiated in a 30 mM EDTA·2Na solution (10 mL) for 6 h; these mixtures were then filtered. The elemental analysis results for Re revealed that the loadings of the obtained solids after the first visible-light irradiation were 0.016 and 0.11 μmol/g, respectively; this suggested that only 4.3%–4.8% of the surface polyoxoanion **2** remained after the first reactions. The obtained solids after the first light irradiation showed values of 500.2 and 169.6 μmol/g after 6 h, respectively, which were 1.2–3 times larger than the values for the first run. TON also reached 62525 and 3084, which were 25–71 times higher than those for the first run, and 2.2–72 times higher than the values obtained for 1-Cs-TiO_2_(2.0) and **1**-Cs-TiO_2_(3.3) in the second run. The ^31^P NMR spectrum in D_2_O of **2** dissolved in aqueous EDTA solution containing TiO_2_ after light irradiation for 6 h showed two signals at −10.61 and −15.36 ppm with ca. 1:2 intensities, which were assigned to [PW_11_O_39_]^7-^ and **2**, respectively. The DR UV-vis spectrum of **2**-Cs-TiO_2_(3.3) after the photoreaction showed significant reduction of the Re^V^ ➔ W^VI^ IVCT and *d*-*d* bands (Figure S2b), suggesting that the molecular structure and/or oxidation state of polyoxoanion **2** were changed under the present reaction conditions. Thus, the catalytic activities of **2**-supported TiO_2_ materials in the second run might be influenced by the conditions of the species formed by the light irradiation and those of **2** in solution and solid state.

**Table 2:** Hydrogen evolution from water catalyzed by **2**-supported TiO_2_ materials under visible light irradiation. ^[a]^

Entry	Catalyst	Reaction time [h]	Recycle	H_2_ [μmol/g]	TON^[b]^
1	**2**-Cs-TiO_2_(0.37)	1	1st run	15.1	–
		6		163.7	885
		1	2nd run	85.5	–
		6		500.2	62525^[c]^
2	**2**-Cs-TiO_2_(2.3)	1	1st run	16.5	–
		6		139.1	121
		1	2nd run	25.0	–
		6		169.6	3084^[c]^
3	**2**-Pt-TiO_2_(0.059)	1	1st run	64.3	–
		6		402.5	13644
		1	2nd run	55.1	–
		6		414.3	85423^[c]^
4	**2**-Pt-TiO_2_(0.19)	1	1st run	59.7	–
		6		141.0	1484
		1	2nd run	76.0	–
		6		352.6	26119^[c]^

[a] Reaction conditions: water (10 mL), catalyst (200 mg), EDTA·2Na (30 mM), light (≥400 nm), 25 ºC.

[b] Turnover number (TON) was calculated as 2[H_2_ evolved (mol/g)] per [Re atoms (mol/g)].

[c] The concentration of Re atoms for **1**-supported TiO_2_ materials after the first visible-light irradiation (≥400 nm) was 0.016, 0.11, 9.7 × 10^-3^, and 0.027 μmol/g, respectively.

For **2**-Pt-TiO_2_(0.059), the amount of H_2_ evolved after 1 and 6 h were 64.3 and 402.5 μmol/g of catalyst, respectively. TON after 6 h was 13644, which was 15–113 times higher than the values for **2**-Cs-TiO_2_(0.37) and **2**-Cs-TiO_2_(2.3). In contrast, **2**-Pt-TiO_2_(0.19) showed significant inactivation; thus, H_2_ evolved 141.0 μmol/g of catalyst (TON = 1484) after 6 h. Only a trace amount of Pt was needed to enhance the catalytic activities; however, inactivation was also caused at higher loadings of Pt species, as reported for the Ru(bpy)_3_^2+^/methyl viologen/EDTA system [31].

The leaching of the surface polyoxoanion **2** into the solution was also determined for **2**-Pt-TiO_2_(0.059) and **2**-Pt-TiO_2_(0.19) under the same reaction conditions. The elemental analysis results of Re revealed that the loadings of the obtained solids after the first visible-light irradiation were 9.7 × 10^−3^ and 0.027 μmol/g, respectively; this suggested that 14%–16% of the surface polyoxoanion **2** remained on the TiO_2_ surface. The recycle experiments of the obtained solids after the first light irradiation showed values of 414.3 and 352.6 μmol/g after 6 h, respectively. TON was 85423 and 26119, which were 6.3–17.6 times higher than the values obtained in the first run.

## 4. Conclusions

The synthesis and full characterization of a Keggin-type mono-rhenium(V)-substituted polyoxoanion are presented. We successfully obtained black-purple crystals of the dimethylammonium salt [Me_2_NH_2_]_4_[PW_11_Re^V^O_40_] (**Me_2_NH_2_-2**) by treating [Re^IV^Cl_6_]^2-^ with a mono-lacunary Keggin polyoxoanion. Compound **Me_2_NH_2_-2** was characterized by X-ray structure analysis, elemental analysis, TG/DTA, UV-vis absorption, FTIR, and solution ^31^P NMR spectroscopy. The crystal structure of **2** revealed a monomeric structure with overall *T_d_* symmetry; however, the rhenium(V) site was not determined due to the high symmetry.

The Dawson- and Keggin-type rhenium(V)-containing polyoxoanions [O{Re^V^(OH) (α_2_-P_2_W_17_O_61_)}_2_]^14-^ (**1**) and **2** were supported onto a TiO_2_ surface by precipitation methods using CsCl and Pt(NH_3_)_4_Cl_2_. With these **1**- and **2**-supported TiO_2_ materials, hydrogen evolution from water in the presence of EDTA·2Na under visible light irradiation (≥400 nm) was achieved. The results for the photoreactions showed the following. (1) The catalytic activities of **2**-supported TiO_2_ materials were higher than those of **1**-supported materials at similar loadings. (2) Significant leaching of the surface polyoxoanions **1** and **2** was observed after the first light irradiation, and the polyoxoanion leached into the solution showed hydrogen evolution from water in the presence of TiO_2_. (3) The stability of **1** in both solution and solid state was higher than that of **2** under the light irradiation. (4) Surface Pt species enhanced the catalytic activities; however, inactivation was also observed at higher loadings. (5) The catalytic activities in the second run were higher than those in the first run for both **1**- and **2**-supported TiO_2_ materials, regardless of the grafting methods used for CsCl and [Pt(NH_3_)_4_]Cl_2_.

## Supporting Information

Figures S1–S5 can be downloaded online at can be downloaded online at http://www.mdpi.com/1996-1944/3/2/897/s1.
