# Supplementary material for: EDTA-Reduction of Water to Molecular Hydrogen Catalyzed by Visible-Light-Response TiO2-Based Materials Sensitized by Dawson- and Keggin-Type Rhenium(V)-Containing Polyoxotungstates
Source: Materials (Basel). 2010 Feb 2;3(2):897–917. doi: 10.3390/ma3020897 (PMC5513514; doi:10.3390/ma3020897)

Supporting information

EDTA-Reduction of Water to Molecular Hydrogen Catalyzed by Visible-Light-Response TiO_2-Based Materials Sensitized by Dawson- and Keggin-Type Rhenium(V)-Containing Polyoxotungstates

Chika Nozaki Kato [1,]*, Kazunobu Hara [1], Masao Kato [1], Hidekuni Amano [1], Konomi Sato [2], Yusuke Kataoka [2] and Wasuke Mori [2]

[1] Department of Chemistry, Faculty of Science, Shizuoka University, 836 Ohya, Suruga-ku, Shizuoka 422-8529, Japan

[2] Department of Chemistry, Faculty of Science, Kanagawa University, Tsuchiya 2946, Hiratsuka, Kanagawa 259-1293, Japan

Figure S1. Diffuse reflectance UV-vis spectra in the visible region. (a) **1**-Cs-TiO_2(2.0). (b) **1**-Cs-TiO_2(3.3).

(a)

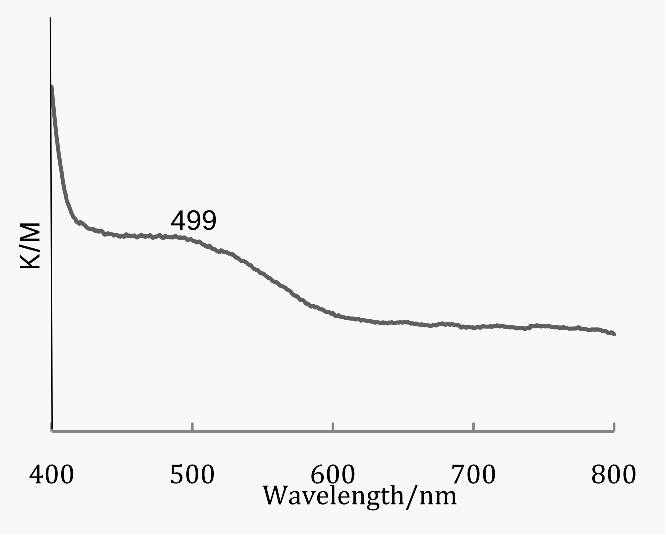

(b)

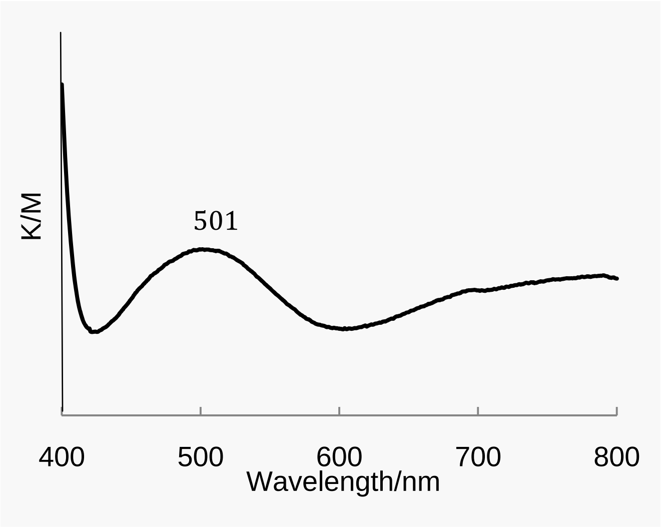

Figure S2. Diffuse reflectance UV-vis spectrum in the visible region. (a) As-prepared **2**-Cs-TiO_2(2.3). (b) After photoreactions.

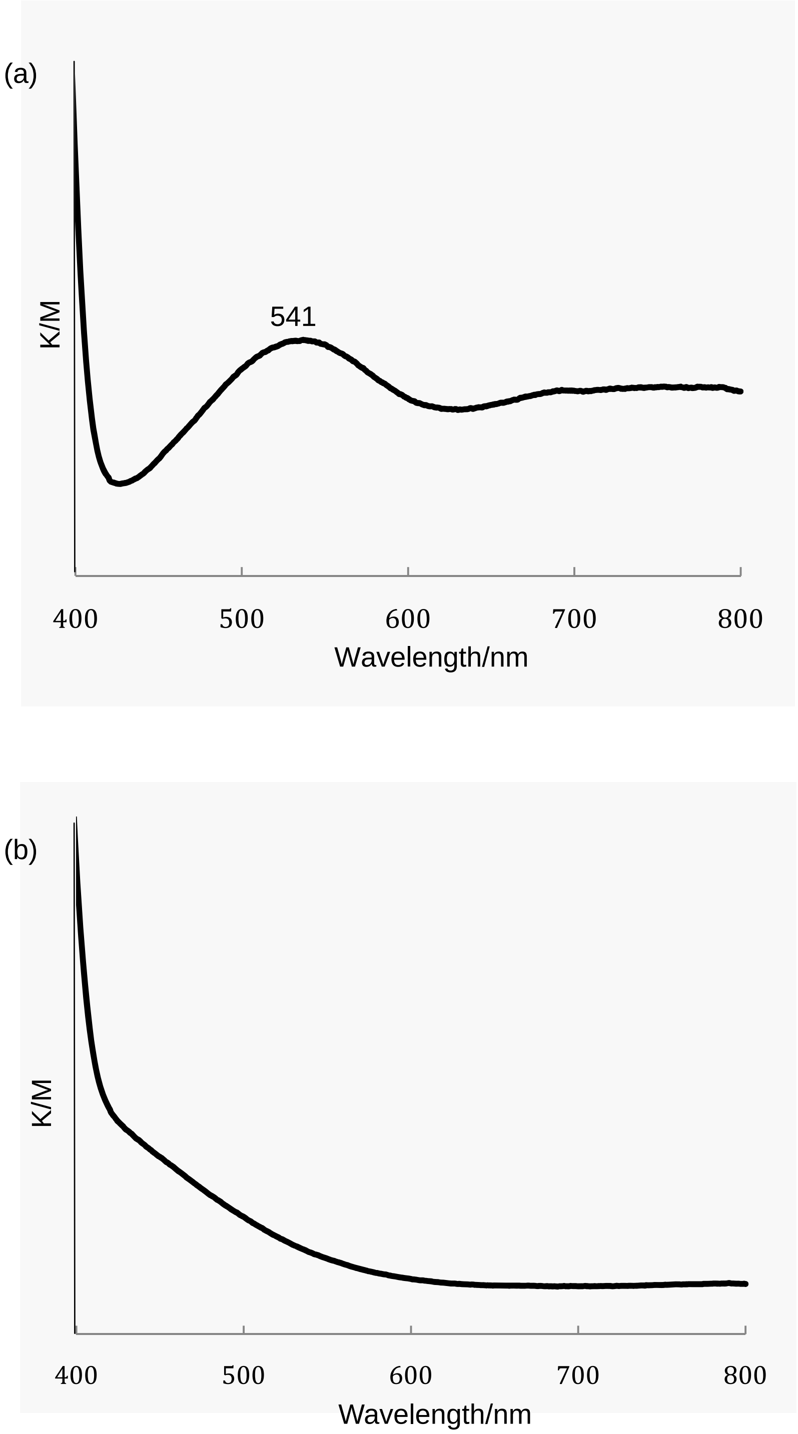

Figure S3. Diffuse reflectance UV-vis spectra in the visible region. (a) **1**-Pt-TiO_2(1.6). (b) **1**-Pt-TiO_2(3.9). (c) **1**-Pt-TiO_2(5.6).

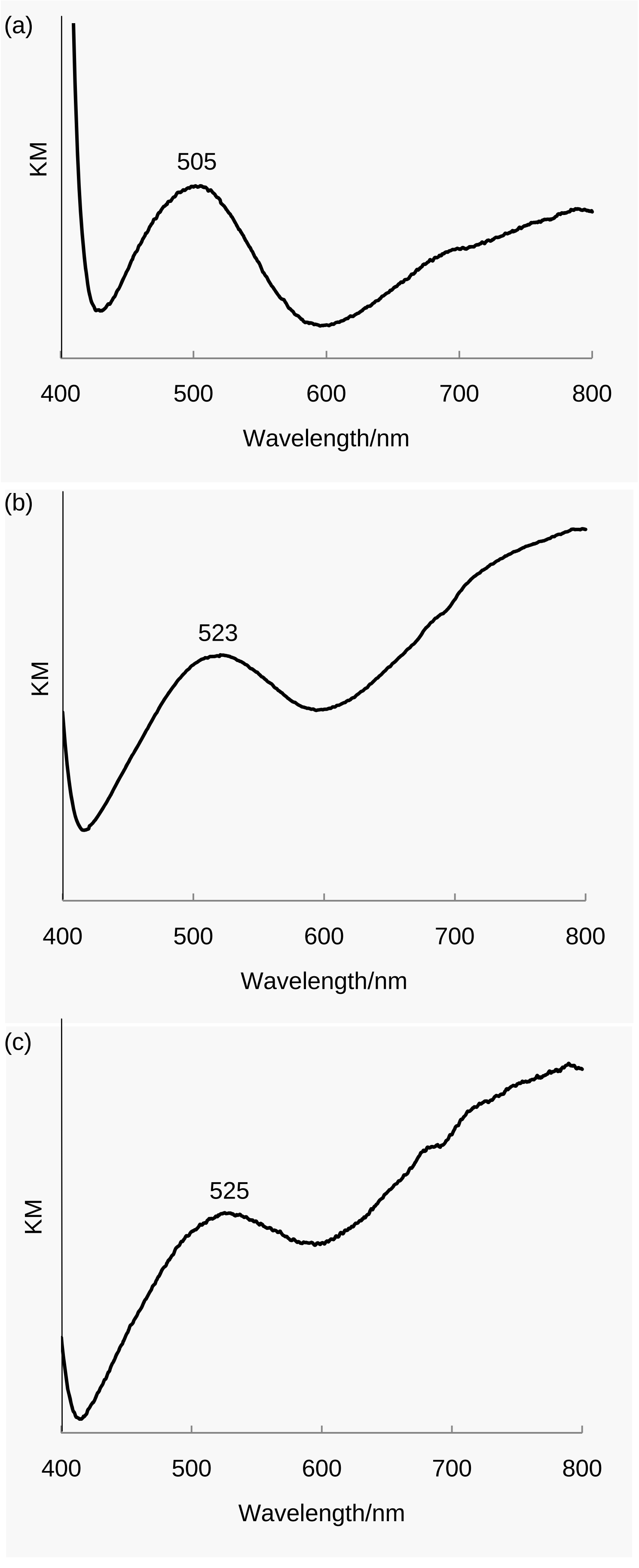

Figure S4. Diffuse reflectance UV-vis spectra in the visible region for (a) **1**-Cs-TiO_2(2.0) and (b) **1**-Cs-TiO_2(3.3) after the photoreaction.

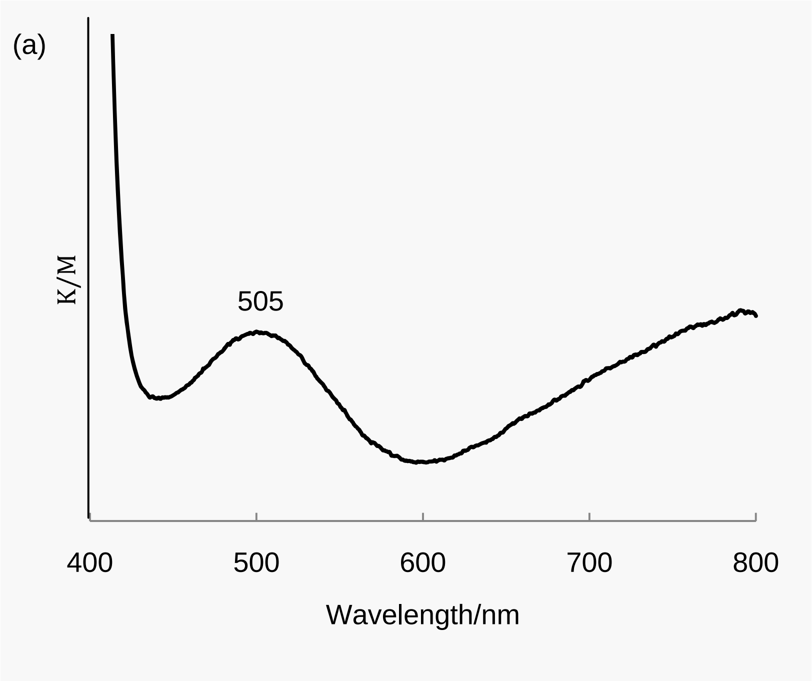

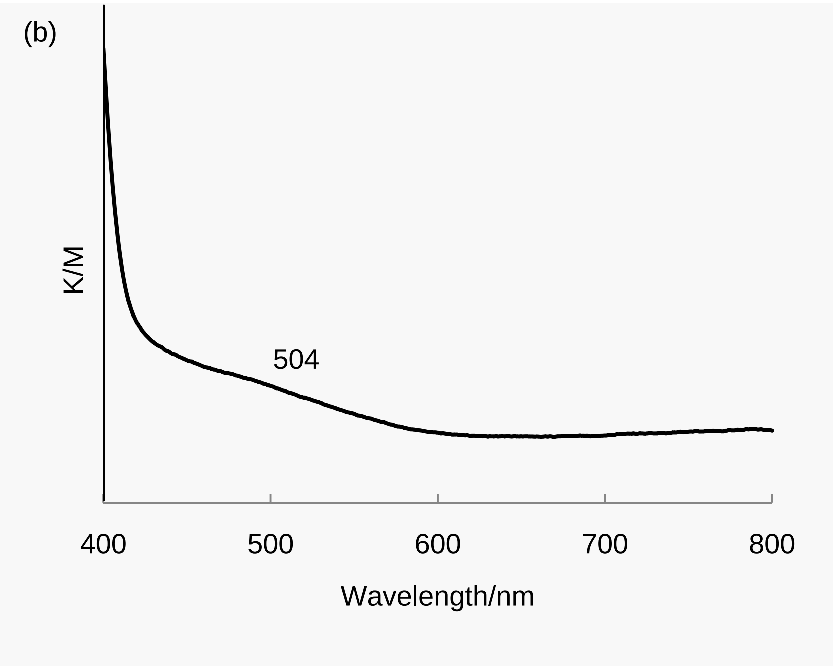

Figure S5. Diffuse reflectance UV-vis spectra for (a) **1**-Pt-TiO_2(1.6), (b) **1**-Pt-TiO_2(3.9), and (c) **1**-Pt-TiO_2(5.6) after the photoreaction.

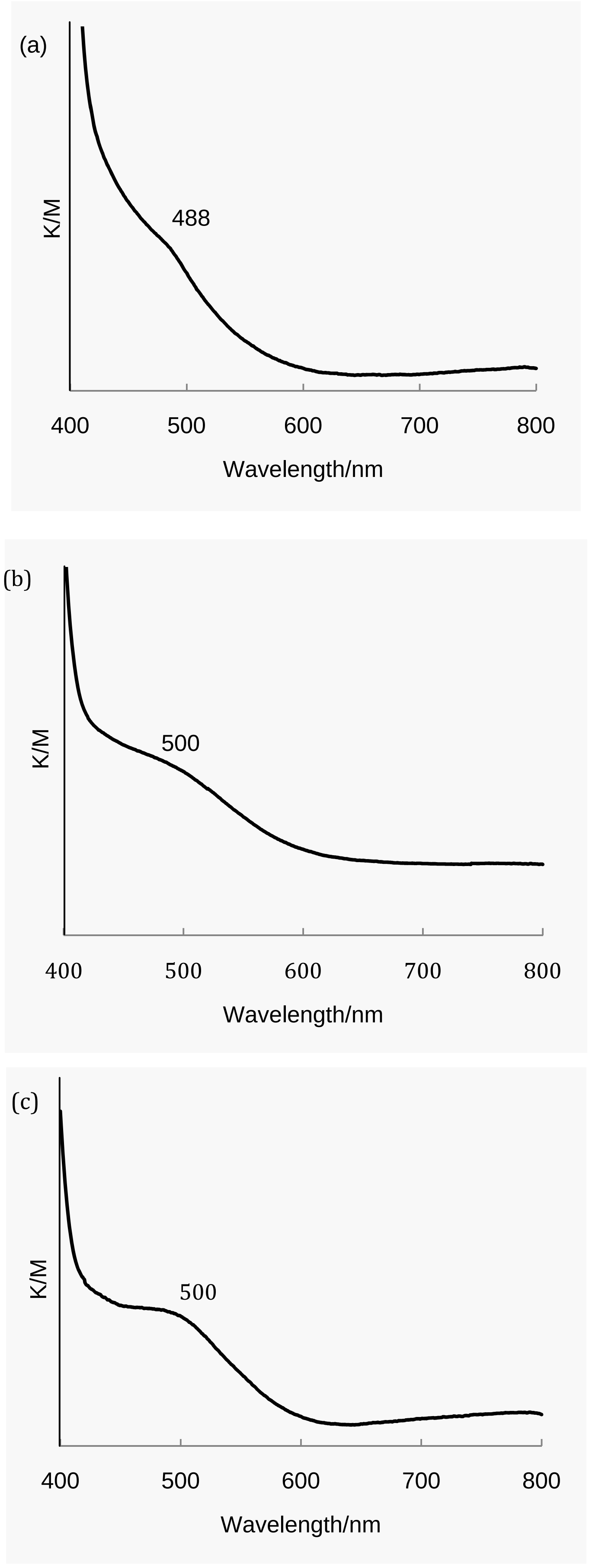

Supplement: Supplementary file 1 [file materials-03-00897-s001.pdf]
